# The role of the dorsal hippocampus in two versions of the touchscreen automated paired associates learning (PAL) task for mice

**DOI:** 10.1007/s00213-015-3949-3

**Published:** 2015-05-13

**Authors:** Chi Hun Kim, Christopher J. Heath, Brianne A. Kent, Timothy J. Bussey, Lisa M. Saksida

**Affiliations:** Department of Psychology, MRC/Wellcome Trust Behavioural and Clinical Neuroscience Institute, University of Cambridge, Downing St, Cambridge, CB2 3EB UK

**Keywords:** Mouse, Paired-associate learning, Hippocampus, Touch screen operant chamber

## Abstract

**Rationale:**

The CANTAB object-location paired-associate learning (PAL) test can detect cognitive deficits in schizophrenia and Alzheimer’s disease. A rodent version of touch screen PAL (dPAL) has been developed, but the underlying neural mechanisms are not fully understood. Although there is evidence that inactivation of the hippocampus following training leads to impairments in rats, this has not been tested in mice. Furthermore, it is not known whether *acquisition*, as opposed to performance, of the rodent version depends on the hippocampus. This is critical as many mouse models may have hippocampal dysfunction prior to the onset of task training.

**Objectives:**

The objectives of this study are to examine the effects of dorsal hippocampal (dHp) dysfunction on both performance and acquisition of mouse dPAL and to determine if hippocampal task sensitivity could be increased using a newly developed *context-disambiguated PAL* (cdPAL) paradigm.

**Methods:**

In experiment 1, C57Bl/6 mice received post-acquisition dHp infusions of the GABA agonist muscimol. In experiment 2, C57Bl/6 mice received excitotoxic dHp lesions prior to dPAL/cdPAL acquisition.

**Results:**

Post-acquisition muscimol dose-dependently impaired dPAL and cdPAL performance. Pre-acquisition dHp lesions had only mild effects on both PAL tasks. Behavioural challenges including addition of objects and degradation of the visual stimuli with noise did not reveal any further impairments.

**Conclusions:**

dPAL and cdPAL performance is hippocampus-dependent in the mouse, but both tasks can be learned in the absence of a functional dHp.

## Introduction

Object-location paired-associate learning (PAL) as tested in the touch screen CANTAB battery is highly sensitive to impairment in Alzheimer’s disease (AD) and schizophrenia yet is not disrupted in conditions such as depression (Swainson et al. 2001), thereby conveying good task specificity. In AD, PAL is highly sensitive to disease progression (Fowler et al. 2002), and a short tablet computer-based assessment tool based on this task has recently been made available to general practitioners in the UK to help with dementia detection (Todd 2013; Cambridge Cognition 2014). In schizophrenia, PAL is impaired not only in patients with chronic schizophrenia but also in individuals at risk of disease (Wood et al. 2002; Barnett et al. 2005). In addition, PAL performance has been shown to be related to the global severity and functional independence of the patients (Barnett et al. 2005; Aubin et al. 2009).

A version of object-location PAL for rodent models of disease (dPAL) has been developed in the touch screen operant behavioural system. To date, it has been validated in rats (Talpos et al. 2009; McAllister et al. in this issue) and also used to assess cognitive deficits in genetically modified mice (Bartko et al. 2011a; Coba et al. 2012; Nithianantharajah et al. 2013). The full translational potential of this task was recently demonstrated in a study showing parallel cognitive impairments in both mice (on dPAL) and humans (on CANTAB PAL) with mutations in the *Dlg2* gene, which has been associated with schizophrenia (Nithianantharajah et al. 2013). More recently, these same individuals have been shown to be impaired on the mouse version of the task (dPAL; Nithianantharajah et al., under revision).

Although the evidence so far is promising, the neural underpinnings of PAL are not completely understood. A core feature of both CANTAB PAL and dPAL is the requirement to associate objects with their matched locations on a touch screen. As the hippocampus is known to be an area in which object and spatial information converges (Eichenbaum et al. 2012), it is likely that this region is critical for this process. Work with non-touch screen tasks indicates that the hippocampus is important generally in object-location memory (Komorowski et al. 2009; Barker and Warburton 2011), and current evidence suggests that this is the case for PAL as well. For example, in a human fMRI study, de Rover et al. (2011) found bilateral activation of the hippocampus during the encoding phase of CANTAB PAL. Participants with memory deficits showed decreasing hippocampal activation with increasing memory load, whereas normal controls showed the opposite pattern. In addition, Kéri et al. (2012) showed that impairment on CANTAB PAL was correlated with hippocampal volume loss in both schizophrenia and mild cognitive impairment. The hippocampus also appears to be involved in the rat version of the task as acute inactivation after dPAL acquisition has been shown to impair performance (Talpos et al. 2009).

Increasingly, mice are the species of choice for disease models and the study of the relationship between genes and behaviour. However, the hippocampal dependency of dPAL performance has not been tested in this species, thereby making the neurobiological interpretation of any observed behavioural alterations challenging. Similarly, many mouse disease models may exhibit hippocampal dysfunction prior to the onset of task training, and so, whether *acquisition*, as opposed to performance, of dPAL depends on the hippocampus is also a critical unanswered question. Finally, while lidocaine-mediated inactivation of the rat dorsal hippocampus was found to impair post-acquisition dPAL performance (Talpos et al., 2009), the magnitude of this impairment was moderate. It is possible that altering the spatial demands of the task, or using different methods of inactivating the hippocampus, might yield more robust effects.

As part of the NEWMEDS consortium, we have been working on the identification and validation of a translational touch screen test battery for preclinical studies in schizophrenia (summarised in Hvoslef-Eide et al., this issue). One of the key tests in the battery is dPAL, and resolution of the unresolved issues relating to the mouse version of this paradigm is of critical importance. Therefore, in this study, we investigated the effects of hippocampal inactivation using muscimol at two different doses to determine if dPAL in this species is sensitive to hippocampal inactivation, as it is in rats. We also examined if acquisition of the task is hippocampus-dependent in mice by making permanent excitotoxic hippocampal lesions prior to PAL acquisition. In parallel with these experiments, we developed a new version of mouse PAL (*context-disambiguated PAL* (cdPAL)), in which visual stimuli were presented in two physically separated areas in the touch screen chamber (distinct yet similar enough to tap the putative ‘pattern separation’ functions of the hippocampus), to test whether presenting stimuli in two contexts would increase task hippocampal sensitivity.

## Methods and materials

### Subjects

See individual experiment sections. All experimentation was conducted in accordance with the UK Animals (Scientific Procedures) Act, 1986.

### Apparatus

**Fig. 1:**
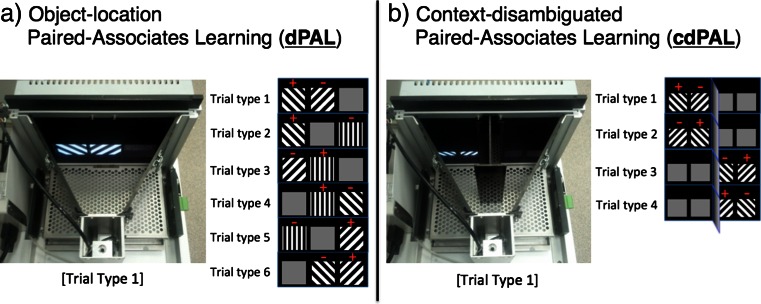
Photographs of the touch screen operant chamber system and depictions of trial types used in the **a** object-location paired-associates learning (dPAL) and **b** context-disambiguated paired-associate learning (cdPAL) tasks. In the trial type figures, *positive sign* denotes the correct object and *negative sign* denotes the incorrect object in each trial type. In cdPAL, a vertical barrier is inserted to divide the arena into two spatial contexts with a connecting central zone at the rear of the chamber

Mice were tested using touch screen operant chambers (Campden Instruments Ltd., UK) which have been described in previous reports (Horner et al. 2013). In brief, the testing was conducted in a trapezoidal-shaped operant chamber composed of a metal floor, a reward delivery magazine, a touch screen, two infrared (IR) beams for motor activity detection and black Perspex side-walls (see Fig. 1). The chamber was housed inside a sound- and light-attenuating box with a house light, a tone generator, a ventilating fan and an IR camera. ABET software provided by Campden Instruments controlled the system and collected data. For the current experiment, two types of black Perspex masks, placed over the touch screen, were used: one with three response windows (each square 7 × 7 cm) (for dPAL) and the other with four response windows (each square 4 × 4 cm) (for cdPAL). For cdPAL, a custom-made black Perspex barrier was placed inside the chamber to divide the arena into two spatial contexts (see Fig. 1b). Liquid reward (strawberry milkshake; Yazoo, FrieslandCampina, Ltd.) was provided to motivate task performance.

### Behavioural procedures

Pre-training prior to PAL task training has been described previously (Horner et al. 2013; Oomen et al. 2013). In brief, mice were acclimatised to the animal facility under a 12-h light cycle (lights off at 7 a.m.) for at least 7 days following delivery. After this period, food was restricted to maintain 85–90 % of free feeding body weight. Behavioural training began with a 20-min operant chamber habituation session. Following habituation, mice were trained to touch a white square stimulus presented pseudo-randomly in one of the response windows to obtain a reward in the magazine (criterion: collection of 36 rewards within 60 min). Animals were then trained to break the IR beam near the reward magazine to initiate trials (criterion: collection of 48 rewards within 45 min). In the final stage of pre-training, a touch to windows without a stimulus resulted in a 5-s time-out signalled by house light illumination. This was followed by a 5-s intertrial interval (ITI) and correction trials with the stimulus presentation in the same response window until the mouse made a correct response. Reward collection for a correct touch was followed by a 15-s intertrial interval (ITI). When all mice completed 48 trials within 30 min at over 80 % correct for two consecutive sessions, they were moved onto either dPAL or cdPAL training.

We modified the previously described dPAL training procedure (Talpos et al. 2009; Bartko et al. 2011b; Horner et al. 2013) for the current study. Line stimuli were used which we have found in pilot studies to yield reduced performance variability compared to ‘shape’ stimuli previously used with this task. As shown in Fig. 1a, one of the six possible trial types was pseudo-randomly selected and presented on the screen. For each trial type, one visual stimulus was presented in its correct location, the second visual stimulus was presented in one of its two incorrect locations, and the third location remained blank. Each trial type (except for correction trials) was presented an equal number of times and was not repeated on more than two consecutive trials. A correct choice was followed by reward delivery, a 15-s ITI and then the next trial. An incorrect choice was followed by a 5-s time-out and a 5-s ITI, after which correction trials continued until the correct choice was made. Correction trials were excluded from total trial count and are not included in the calculation of percent correct, as they likely reflect constructs such as inhibition of prepotent responses (e.g. side biases) that are not the focus of interest (object-location paired-associate learning). During performance testing, correction trials were not given. This is because in a pilot study, on infusion days, we found that some mice did not complete all trials within a session. Furthermore, the purpose of correction trials is to reduce unwanted side biases, and by the performance stage, the task is well learned and side biases more or less extinguished. The maximum number of trials per session started at 24 and was gradually increased up to 96. The session finished either when the maximum trial number was reached or when 60 min had passed.

cdPAL is a novel variant of the touch screen PAL task in which the test arena is divided into two spatial contexts by a black Perspex barrier (see Fig. 1b). The barrier projected 10 cm from the screen leaving a space behind it 7.5 cm from the magazine through which the mouse could transition from context to context. Whether an object was designated correct or not was dependent on the spatial context in which it was presented. For instance, left diagonal was correct on the left side of the chamber (trial type 1 and 2), but right diagonal was correct on the right side of the chamber (trial type 3 and 4). Other procedures were the same as in dPAL training.

### Hippocampus cannula implantation

Mice were anaesthetised with isoflurane gas and placed in a stereotaxic frame (David Kopf Instruments, Tujunga, California, USA). Intraperitoneal (IP) meloxicam (Metacam, Boehringer Ingelheim, Bracknell, UK; 1 mg/10 ml in phosphate-buffered saline (PBS), 0.1 ml per 10 g body weight) was given for perioperative pain control. After exposure of the dorsal skull, two holes were made at the following coordinates: anterior-posterior (AP), −1.7 and medial-lateral (ML), ±1.5 (in mm, from bregma). Through the holes, a double-guide cannula (C232GC, 22 gauge, Plastics One, Roanoke, VA) inserted with a dummy cannula (C232DC; Plastics One) was implanted aiming at the bilateral dorsal hippocampus (dHp) and secured to the skull with dental cement (Super-Bond C&B, Sun Medical Ltd.) and screws. Finally, the incision of the scalp was sutured around the cannulae and the mouse was observed until fully mobile in a recovery chamber (maintained at 30 °C). Before returning to their home cage, mice were singly housed overnight. Following surgical recovery, mice were provided with ad libitum food and water for at least 7 days before behavioural procedures and food restriction recommenced.

### Drug preparation and cannula infusion

Muscimol hydrobromide (Sigma-Aldrich, UK) was dissolved in physiological saline to the target concentrations of 0.1 and 0.2 μg/μl (chosen based on a pilot study: data not shown). To habituate mice to the infusion procedure, two mock infusion sessions were given. In the first mock session, the experimenter gently restrained the mouse and replaced a dummy cannula with an injector cannula (C232I; intrahippocampal: 26 gauge, extending 0.88 mm beyond the 1.5-mm guide cannulae). After 1 min, the injector was removed and the dummy cannula replaced. In the second mock session, physiological saline was infused bilaterally (0.25 μl/side at a rate of 0.33 μl/min) using a syringe pump (Harvard Apparatus) with two 10-μl Hamilton syringes connected to the injector cannula. The infusion volume and rate were chosen based on a previous paper (Misane et al. 2013) in which spread was restricted to the dorsal hippocampus. In the drug infusion sessions, the procedures were the same as the second mock infusion, but saline, muscimol 0.1 μg/μl and 0.2 μg/μl were infused in every mouse in a pseudo-random order. The injection cannula was left in situ for 1 min after the infusion to help further diffusion of the drug. PAL testing started 30 min after the infusion. At least 48 h was given between infusions to allow for washout of the drug and recovery of baseline performance to >75 % correct.

### Hippocampus lesion surgery

Mice were anaesthetised with isoflurane gas and placed in a stereotaxic frame (David Kopf Instruments, Tujunga, California, USA). Intraperitoneal (IP) meloxicam, described above, was given for perioperative pain control. After exposure of the dorsal skull, an imaginary line connecting lambda and bregma was aligned with a horizontal plane of the frame. In the hippocampus lesion group, four holes were drilled at the following coordinates: [site 1, 2] AP −1.7; ML ±1.0, [site 3, 4] AP −2.3; ML ±1.7 (in mm, AP and ML from bregma). A 5-μl Hamilton syringe fitted with 33-gauge needle was lowered vertically 1.9 mm from the surface of the skull for injections through the holes. For each animal, four injections of 10 mg/ml NMDA (Sigma, UK) in PBS solution were made at a rate of 0.1 μl/min for a volume of 0.1 μl for sites 1 and 2 and 0.2 μl for sites 3 and 4. After each injection, the needle was left for 4 min for diffusion of the drug. For the sham lesion group, four holes were made at the same coordinates and the same needle without NMDA was lowered through the cortex but not into the hippocampus. After scalp suture, mice were kept in a recovery chamber (maintained at 30 °C) until fully mobile. Before returning to their home cage, the mice were individually housed overnight. The mice were given ad libitum access to drinking water and food until they had regained stable weights. When seizures were observed during the post-surgery recovery period, IP injections of diazepam (10 mg/10 ml in ethanol and PBS, 0.1 ml per 10 g body weight) were given.

### Histology

At the end of behavioural testing, mice were anaesthetised by an IP injection of Dolethal (Vetoquinol UK Ltd., Buckinghamshire, UK) and transcardially perfused with PBS, followed by 10 % neutral buffered formalin (NBF). Brains were removed, post-fixed in 10 % NBF at 4 °C overnight and then soaked in 30 % sucrose in PBS until they sank. Using a freezing microtome, 60-μm coronal sections were taken covering the full hippocampus. Every fourth section was stained with cresyl violet and examined using a light microscope.

### Data analysis

All data were checked for normality by the Shapiro-Wilk test and for homogeneity of variance by Levene’s test and analysed using independent samples *t* tests or the Mann-Whitney *U* tests as appropriate. Data with within-subject factors were subjected to repeated measures ANOVA (rmANOVA). Violation of sphericity was corrected by the Greenhouse-Geisser (GG) method. Motor activity was measured by the number of IR beam breaks near the magazine per minute. For latencies, median latency was taken for a given session, rather than a mean, to minimise the effects of anomalously high latencies among multiple trials within a session. All statistical analyses were conducted with SPSS version 22.

## Experiment 1: post-acquisition hippocampal inactivation in two versions of the PAL task

In this experiment, we first investigated the effects of hippocampal inactivation on dPAL in mice to test whether the findings in rats (Talpos et al. 2009) would replicate in mice. Second, we developed a new context-dependent cdPAL task, in which visual stimuli were presented in two separated areas in the touch screen chamber. This was done to test whether the incorporation of more explicit contexts would increase sensitivity to hippocampal inactivation.

### Methods

Twenty-four male C57Bl/6 mice (Harlan, Bicester, UK) started training for dPAL (*N* = 12) and cdPAL (*N* = 12) at 8–9 weeks old. Detailed procedures are described in the general methods section. To prevent overtraining of fast learners, when a mouse met the criterion of over 75 % correct for two consecutive days, it was rested until all the other mice caught up. Reminder sessions were given to all rested mice every Monday and Thursday. In cases where performance in the sessions did not reach 75 % correct, further daily sessions were given until criterion was achieved. Following bilateral hippocampal cannulation surgery, infusions were conducted once all the mice reached the criterion of 75 % correct for two consecutive days. Following completion of behavioural testing, histology was done to check the cannula placements. Statistical analysis for % correct was performed using PAL task type, i.e. dPAL or cdPAL, as a between-subject factor. However, due to the different physical features of the two tasks, i.e. barrier and mask, the analysis for motor activity and latencies was done separately for each. Two mice were excluded from this study: one from the dPAL group died during surgery, and one from the cdPAL group was not able to meet the acquisition criterion.

### Results

**Fig. 2:**
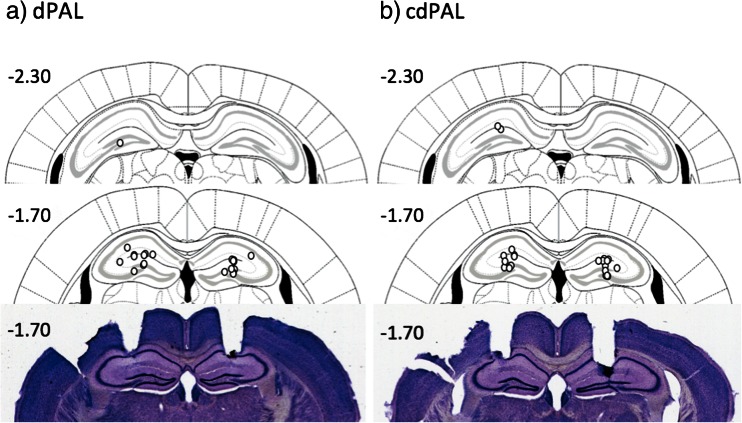
Schematic drawing of the location of cannula infusion sites and photographs of two coronal sections. Each *circle* represents the approximate cannula placement. The *numbers* are distance in millimetre in relation to bregma

**Fig. 3:**
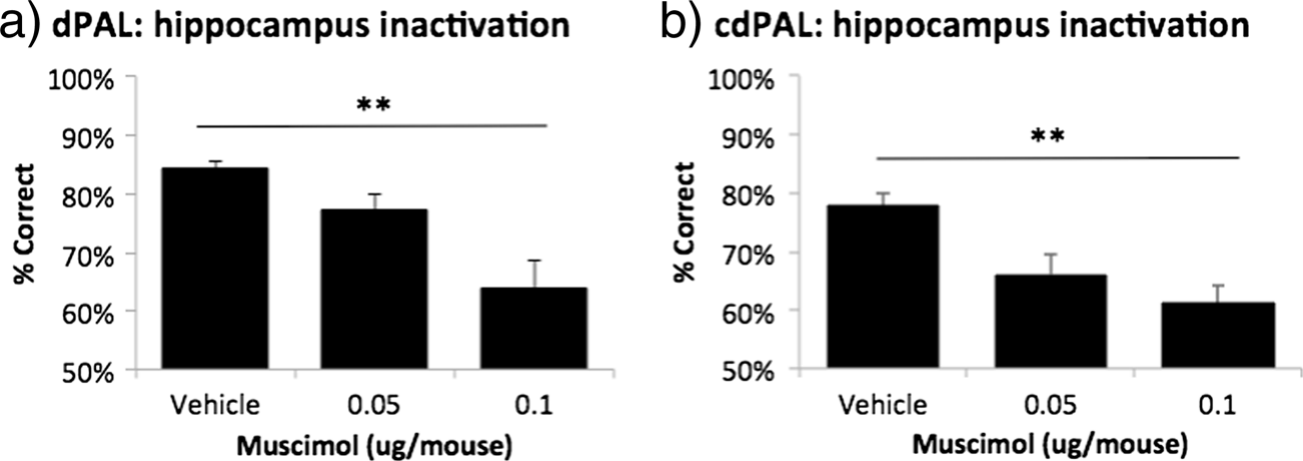
Effects of post-acquisition muscimol infusion into the hippocampus **a** in dPAL and **b** in cdPAL. Data are presented as mean + standard error of the mean (SEM). **p < 0.005

Histological analysis showed that all cannula tips were placed in the dHp (see Fig. 2). The number of sessions taken to reach criterion was not different between the two PAL tasks (*t*(20) = 0.0, ns; *M* 16.0, *SD* 3.77 for dPAL, *M* 16.0, *SD* 3.41 for cdPAL). Infusion of muscimol into the dHp caused a significant dose-dependent impairment in PAL performance (main effect of dose: *F*(1.52,30.41) = 25.28, *p* < 0.001; see Fig. 3). This effect was not different between the two versions of PAL (task by dose interaction: *F*(1.52,30.41) = 1.34, *p* = 0.271).

According to the number of beam breaks recorded, muscimol did not affect motor activity in either task (dPAL: *F*(2,20) < 1, ns, cdPAL: *F*(2,20) = 1.31, *p* = 0.292; see Table 1); however, there was a trend towards increasing response latency in both tasks (dPAL: *F*(1.02,10.20) = 4.68, *p* = 0.055, cdPAL: *F*(1.16,11.58) = 3.68, *p* = 0.076). In the cdPAL task, reward collection latency was significantly larger after muscimol infusion (*F*(2,20) = 8.04, *p* = 0.003) and pairwise comparison with Bonferroni correction showed that the difference was between vehicle and high dose (0.1 μg/mouse) but not in any other comparison (vehicle vs. low dose *p* = 0.213, vehicle vs. high dose *p* = 0.014, low vs. high dose *p* = 0.151). In the dPAL task, the same pattern was observed, but it did not reach statistical significance (*F*(1.21,12.08) = 2.17, *p* = 0.165).

**Table 1:** Effects of intra-hippocampal muscimol (μg/mouse) on motor activity and latencies

Task	Vehicle	0.05	0.1
Motor activity			
dPAL	6.61 ± 0.54	7.43 ± 0.83	7.34 ± 0.55
cdPAL	7.04 ± 0.25	7.82 ± 0.39	7.19 ± 0.51
Response latency			
dPAL	2.12 ± 0.16	2.30 ± 0.17	4.68 ± 1.08
cdPAL	2.19 ± 0.11	3.24 ± 0.50	5.04 ± 1.40
Reward collection latency			
dPAL	1.28 ± 0.12	1.31 ± 0.13	1.70 ± 0.25
cdPAL	1.38 ± 0.05	1.52 ± 0.09	1.79 ± 0.12*

Motor activity indicates the number of IR beam breaks near the magazine per minute. Data are presented as mean ± standard error of the mean (SEM)

*p < 0.05 compared to vehicle

## Experiment 2: pre-acquisition hippocampal lesion in two versions of the PAL task

Experiment 1 showed that the hippocampus plays an important role for both dPAL and cdPAL performance when the task is acquired with an intact hippocampus. However, in some mouse models, hippocampal dysfunction may be present prior to PAL task training. To test whether acquisition was sensitive to hippocampus dysfunction, we assessed PAL acquisition in mice with permanent neurotoxic hippocampal lesions. Following acquisition, mice were further challenged by the introduction of additional objects to be mapped onto the three locations in dPAL or within the two spatial contexts in cdPAL and also by adding noise to the stimuli.

### Methods

A new cohort of 48 male C57Bl/6 mice (Harlan, Bicester, UK) was assigned to the two PAL tasks: dPAL  (*N*= 24) and cdPAL  (*N*= 24). Twelve mice in each group received either hippocampal lesion or sham lesion surgeries as described in the general methods section. Mice were 8–9 weeks old at the time of surgery. After 2 weeks of recovery, behavioural training started and continued until performance stabilised. Since the number of trials per session increased over training, multiple sessions were combined into blocks of approximately 300 trials (range 288–324/block) for graphs and analyses.

**Fig. 4:**
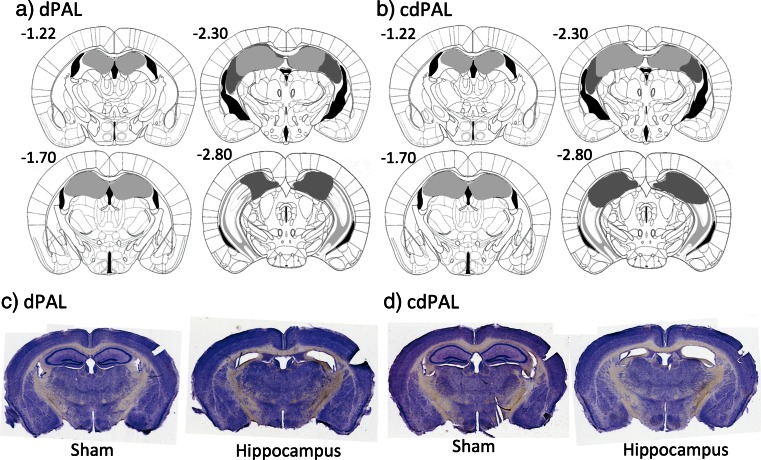
Diagram of the extent of hippocampal lesions and photographs of representative coronal sections. For **a** dPAL and **b** cdPAL, *light grey* represents the smallest lesion and *dark grey* represents the largest. All numbers correspond to distance in millimetre from bregma. Photographs of representative sections corresponding to **−**1.7 mm from bregma are shown for **c** dPAL and **d** cdPAL

Following initial acquisition, the dPAL group was run for two sessions on sPAL, in which an identical object was presented in two locations—one in the correct location and the other in an incorrect location—to test whether hippocampus-lesioned mice were performing the dPAL task by using a conditional rule of the type “if AC, select the left; if CB, select the right” (Talpos et al. 2009; Horner et al. 2013), rather than by an object-location association. If hippocampus-lesioned mice were performing the task using a conditional rule, then switching to sPAL should cause a disproportionate drop in performance in the hippocampus-lesioned group. To see the effects of the barrier after acquisition in cdPAL, it was removed for two sessions. Averaged performance of the last two sessions in dPAL and cdPAL was compared with that of two sessions on sPAL and no-barrier cdPAL, respectively.

After finding that the hippocampal lesion groups in both PAL tasks were performing well above chance level (>80 % correct), mice were further challenged by introducing additional objects to be mapped onto the three locations in dPAL or the two spatial contexts in cdPAL. In the human CANTAB PAL task, this is done by simultaneously increasing both the number of locations and the number of objects (Swainson et al. 2001). However, due to the limited size of the touch screen in rodent chambers, adding more locations would require use of smaller visual stimuli, which has been shown to adversely affect visual discrimination learning (Bussey et al. 2008). Therefore, only the number of objects was increased in this study.

Firstly, new objects were added iteratively to the original stimuli in each task. For dPAL, in which three object-location pairs had been established, we first added a new object which was paired with the left screen response window. In subsequent sessions, we introduced a second new stimulus which was paired with the right response window and, finally, a third new object paired with the central response window. Similarly, for cdPAL, in which two objects were used initially, we iteratively added two further objects (each of which was only correct on one side of the chamber barrier). Three sessions were run after each new object was added. On the first day on a new condition, the performance of mice was often poor and unstable. However, by day 2, the performance and variability were back to normal and were maintained and stable on day 3. Therefore, the averaged performance of the last two sessions was used for analysis.

Secondly, we investigated the learning of new object-location associations in the absence of the previously learnt objects. For this, mice were trained with six new object-location associations in dPAL and four new associations in cdPAL. This is twice the number of objects used in initial task acquisition. Following this, two levels of noise filters (see Fig. 7) were applied to the visual stimuli to reduce their discriminability. Noise was added using an open-source image manipulation programme (GIMP version 2.8.4; downloaded from www.gimp.org) with two steps: Filters > Noise > Hurl > either 25 or 50 % randomisation, then Colors > Desaturate > Average option. Two sessions of each noise filter condition were compared with two sessions of the no-noise condition.

### Result 2.1: histology

Complete bilateral dHp damage, which was defined anterior to −1.7 mm from bregma, was found in all 24 mice of the lesion group (see Fig. 4). Two mice from the lesion group showed bilateral lesions posterior to −2.8 mm from bregma, but all damage was confined to the dorsal region. Bilateral cortical damage along the injection tracks was seen in ten mice: four mice from the dPAL lesion group and six mice from the cdPAL lesion group. Analysis of performance in the last block of trials of initial PAL acquisition (see “Result 2.2: acquisition of two PAL tasks with dorsal hippocampal lesion” section) did not find any significant difference between mice with bilateral cortical damage and other mice in the lesion group (dPAL: *t*(10) = −0.05, *p* = 0.964, cdPAL: *t*(9) = 1.97, *p* = 0.081).

### Result 2.2: acquisition of two PAL tasks with dorsal hippocampal lesion

Acquisition was slower in the hippocampal lesion group during the whole or initial period of training in dPAL and cdPAL, respectively. However, the final performance of the lesion groups in both tasks was over 80 % correct. One mouse from the cdPAL lesion group was excluded from the analysis due to insufficient completion of trials.

**Fig. 5:**
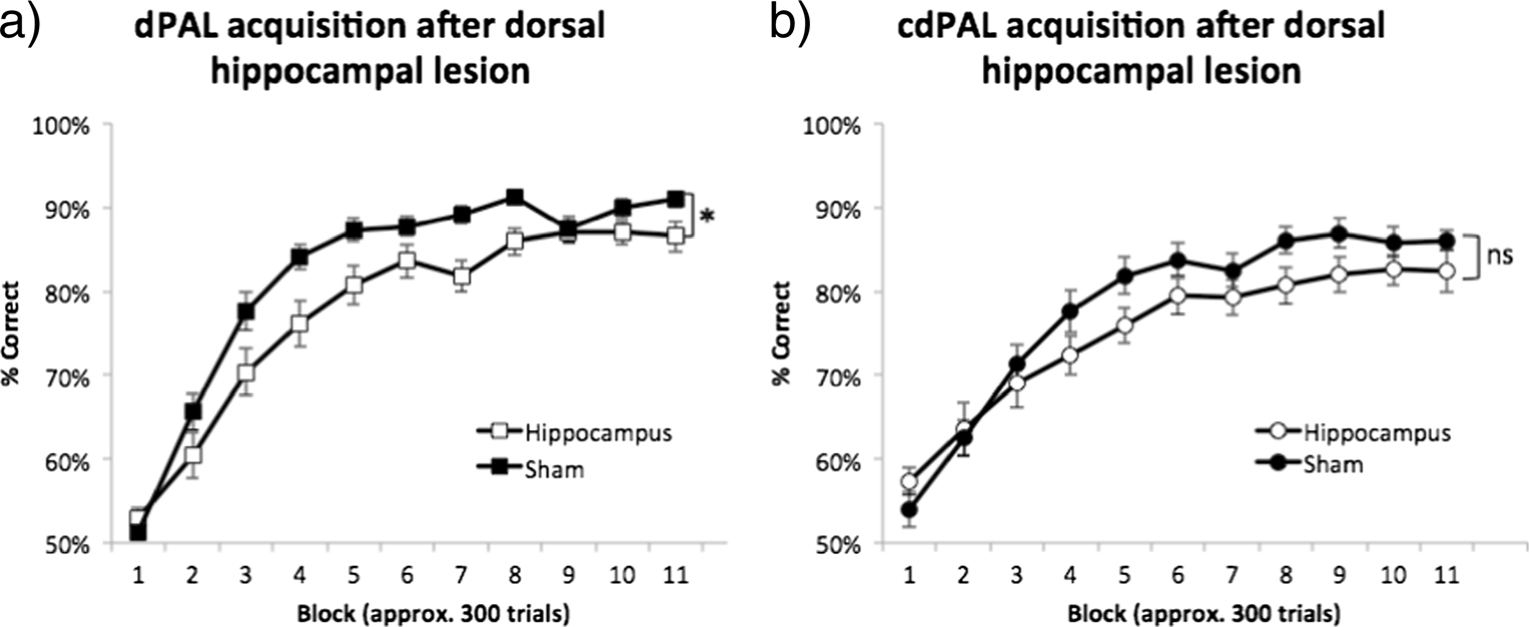
Effects of dorsal hippocampal lesions on the acquisition of **a** dPAL and **b** cdPAL. The number of trials per session increased over training, so multiple sessions were combined into blocks of approximately 300 trials (range 288–324/block). Data are presented as mean ± standard error of the mean (SEM). *p < 0.05 in the main effect of lesion, *ns* not significant

For dPAL, there was a lesion by block interaction (*F*(4.72,103.84) = 3.26, *p* = 0.01; see Fig. 5a) and the hippocampal lesion group performance was significantly lower than the sham group (*F*(1,22) = 3.26, *p* = 0.029). For cdPAL, a lesion by block interaction was present during the first half of training (*F*(3.10,64.99) = 3.26, *p* = 0.016; see Fig. 5b), i.e. block 1–6, but not during the whole training (*F*(3.56,74.77) = 3.26, *p* = 0.107). Also, the performance of the cdPAL lesion group was not significantly different from the sham group during training (*F*(1,21) = 1.72, *p* = 0.204).

Motor activity and latencies were measured using the last block of the initial task acquisition, i.e. block 11. There was a trend towards increased motor activity in the lesion groups, but it was not significant (cdPAL: *t*(21) = 1.87, *p* = 0.075, dPAL: *t*(14.23) = 2.08, *p* = 0.056: see Table 2). Response latency was not different in either task (cdPAL: *t*(21) = 0.356, *p* = 0.725; dPAL: *t*(22) = 0.263, *p* = 0.795). In dPAL, reward collection latency was significantly larger in the lesion group (*t*(15.69) = 2.86, *p* = 0.012) and a similar trend was found in cdPAL (*t*(21) = 1.87, *p* = 0.075).

**Table 2:** Summary of motor activity and latency after dorsal hippocampal lesion

Task	Sham	Hippocampus
Motor activity		
dPAL	7.70 ± 0.46	10.37 ± 1.20
cdPAL	7.80 ± 0.63	9.96 ± 0.99
Response latency		
dPAL	2.02 ± 0.08	2.05 ± 0.08
cdPAL	2.24 ± 0.11	2.31 ± 0.15
Reward collection latency		
dPAL	1.09 ± 0.02	1.24 ± 0.05*
cdPAL	1.18 ± 0.03	1.31 ± 0.06

Motor activity indicates the number of IR beam breaks near the magazine per minute. Data are presented as mean ± standard error of the mean (SEM)

*p < 0.05

### Result 2.3: effects of switching dPAL to sPAL and of removal of the barrier in cdPAL

**Fig. 6:**
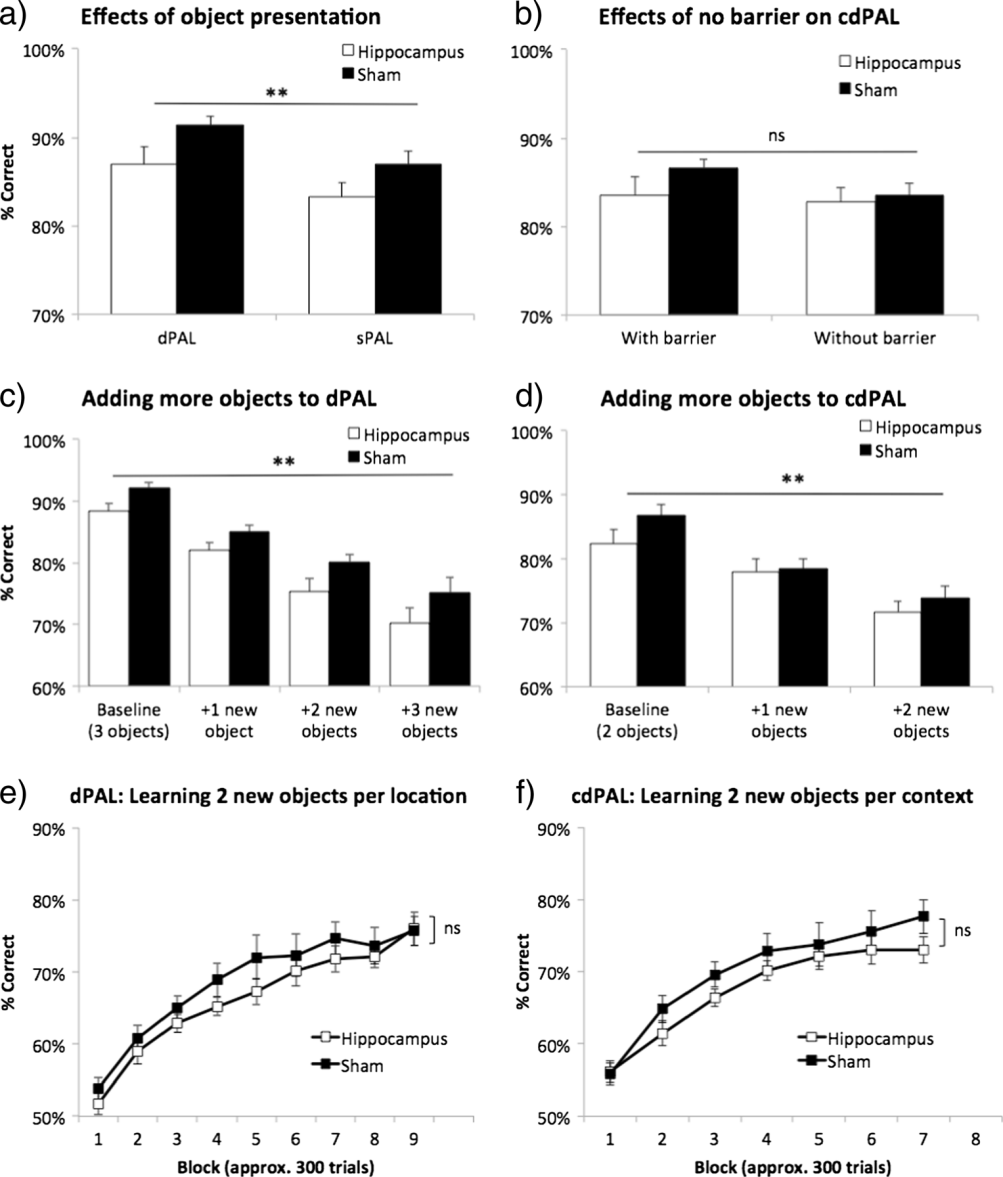
Manipulations to dPAL and cdPAL. First, two identical objects were presented (sPAL) in **a** dPAL and barriers were removed in **b** cdPAL. Second, new objects were added one by one in **c** dPAL and **d** cdPAL. Third, all previously trained stimuli were removed and two novel objects per location/context were trained in **e** dPAL (total six objects) and **f** cdPAL (total four objects). Data are presented as mean + standard error of the mean (SEM). **p < 0.005 in the main effect of manipulation, *ns* not significant

In the dPAL group, changing object presentation in sPAL decreased performance (main effect of task: *F*(1,22) = 11.81, *p* = 0.002; see Fig. 6a), but both lesion and sham groups remained above 80 % correct, and performance of the hippocampus-lesioned mice was not affected disproportionately compared to the sham-lesioned mice. This suggests that the preserved performance in the hippocampus-lesioned mice was not due to these animals solving the task via a conditional rule. The difference between hippocampus-lesioned mice and sham-lesioned mice was maintained (main effect of lesion: *F*(1,22) = 4.78, *p* = 0.04, lesion by task interaction: *F*(1,22) < 1, ns). After the acquisition of cdPAL, removing spatial barriers did not significantly change the performance or the lack of hippocampal lesion effect (main effect of task: *F*(1,21) = 3.79, *p* = 0.059, main effect of lesion: *F*(1,21) < 1, ns, interaction: *F*(1,21) = 1.47, *p* = 0.239; see Fig. 6b). One interpretation is that the barrier served its purpose initially to define the two areas of the chamber for the animal and, thereafter, was not necessary. In fact, we do not know that it was necessary in the first place: we did not attempt a version of the task in which there was no barrier prior to acquisition.

### Result 2.4: increasing cognitive load with more objects in PAL

Both dPAL and cdPAL were sensitive to the addition of new objects to the learnt objects (main effect of object load: *F*(2.14,46.98) = 62.72, *p* < 0.001, in dPAL; *F*(2,44) = 57.95, *p* < 0.001, in cdPAL; see Fig. 6c, d). However, this effect was not dependent on hippocampal lesion (object load by lesion interaction: *F*(2.14,46.98) <1, ns, in dPAL; *F*(2,44) = 1.62, *p* = 0.209, in cdPAL). The same analysis showed a significantly lower performance of the lesion group in dPAL, but not in cdPAL (*F*(1,22) = 5.72, *p* = 0.026; *F*(1,22) = 1.01, *p* = 0.327, respectively).

Removal of all learnt objects and introduction of completely new objects of double the number, i.e. six new objects in dPAL and four new objects in cdPAL, were then used in an attempt to bring out further hippocampal deficits. In both tasks, all mice were able to acquire more stimuli successfully (main effect of block: *p* < 0.001; see Fig. 6e, f), but there was neither a significant lesion by block interaction nor a main effect of lesion in either task (*F* < 1, ns).

### Result 2.5: effects of adding noise to objects on PAL performance

**Fig. 7:**
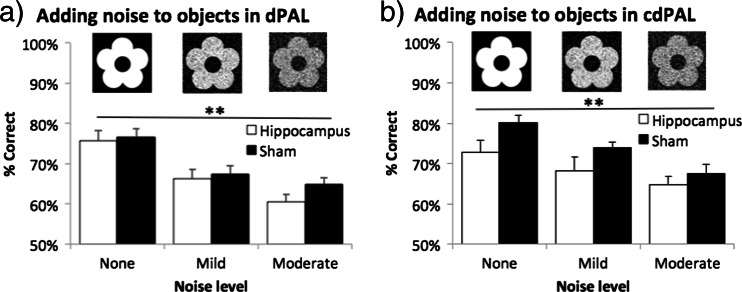
Effects of adding noise to objects in **a** dPAL and **b** cdPAL. Two levels of noise filter were applied to the learnt visual objects to reduce their discriminability. Data are presented as mean + standard error of the mean (SEM). **p < 0.005 in the main effect of noise level

Performance on both dPAL and cdPAL was noise level dependent (main effect of noise level: *p* < 0.001; see Fig. 7a, b). However, there was neither a main effect of lesion (*F*(1,22) < 1, ns, in dPAL; *F*(1,22) = 3.31, *p* = 0.083, in cdPAL) nor a lesion by noise level interaction in any of the tasks (*F* < 1, ns).

## Discussion

The present study had a number of aims. First, we sought to test whether the touch screen dPAL test of object-location learning is sensitive to dHp dysfunction in the mouse, as it is in the rat (Talpos et al. 2009). The development of robust and validated cognitive assays in the mouse is essential to fully capitalise on this species, which is increasingly used in neurobiological investigations and as for models of diseases involving the hippocampus, such as schizophrenia and AD (Papaleo et al. 2012; Hall and Roberson 2012). Second, the previous study of hippocampal dysfunction in dPAL by Talpos et al. (2009) focussed on post-acquisition, asymptotic performance of the task. We sought to test the effects of hippocampal dysfunction occurring prior to acquisition. This question has relevance for use of the task with mouse models in which perturbation of the hippocampus may be present prior to acquisition (e.g. constitutive knock-out or transgenic models). Third, because the effects of hippocampal dysfunction on dPAL previously reported in the rat were moderate, we designed and tested in parallel a new *context-dependent PAL* (cdPAL) task, with a view towards creating a PAL paradigm with increased hippocampal dependency. Lastly, we challenged mice with increasing cognitive load as is done in the human CANTAB PAL (Swainson et al. 2001).

The results were clear. First, performance of dPAL was very sensitive to hippocampal dysfunction in the mouse; infusions of muscimol into the dHp decreased performance in a dose-dependent manner, and percent correct scores dropped from around 80 to around 60 % at the highest dose. This finding demonstrates the utility of dPAL performance as an assay for potential hippocampal dysfunction in the mouse. This also shows that although there are differences between rodent and CANTAB PAL—most notably that rodent PAL is learned over trials rather than very rapidly as in the human version— task performances in the rodent and human versions are similarly dependent on the integrity of the hippocampus (Talpos et al. 2009; de Rover et al. 2011).

Second, pre-acquisition excitotoxic lesions of the dHp had a significant effect on acquisition of dPAL, but this effect was very mild; mice with dHp lesions were able to acquire the task successfully, to above 80 % correct. Furthermore, substituting sPAL (Talpos et al. 2009) for dPAL or increasing the number of objects to be mapped on to the three locations, while decreasing performance levels in both conditions, did not bring out any further hippocampus-dependent impairment. Subsequent acquisition of novel object-location mappings was normal in mice with dHp lesions, as was performance under post-acquisition challenges such as degrading the visual objects with noise. Thus, post-acquisition dysfunction of the dHp strongly affects performance of dPAL, but pre-acquisition dysfunction has little effect on acquisition or subsequent behavioural challenges. This pattern appears to be quite robust: another study has concurrently obtained a very similar pattern of effects using permanent excitotoxic lesions before and after acquisition of dPAL (Delotterie et al. 2015).

Third, the new cdPAL task was also found to be highly sensitive to post-acquisition dysfunction of the hippocampus. As shown in Fig. 3b, infusions of muscimol led to a dose-dependent pattern of impairment highly similar to that seen on dPAL (Fig. 3a). Furthermore, the effect of hippocampal dysfunction on acquisition of cdPAL was similar to that on dPAL, i.e. mild (and in the case of cdPAL, although the initial learning was slower, the main effect of lesion was not significant). Mice with dHp lesions were able to acquire two novel pairs of object-location associations in cdPAL normally, and as in dPAL, post-acquisition challenges such as degradation of the visual objects with noise did not bring out an impairment. Thus, the pattern of effects of dHp dysfunction across performance, acquisition and challenges was highly similar in cdPAL and dPAL. Thus, whereas cdPAL may provide an alternative object-location test to dPAL which may rely on different neural mechanisms, in terms of dHp involvement, the two tasks appear equivalent.

An outstanding question is why, in both dPAL and cdPAL, hippocampus dysfunction induced following acquisition strongly impairs performance, while dysfunction induced prior to acquisition has little effect on acquisition of these tasks. One possibility is that when the hippocampus is intact, the task is learnt in a hippocampus-dependent way—such that post-acquisition manipulations of the hippocampus affect performance—but in the absence of a functional hippocampus, the task can be learnt using an alternative, hippocampus-independent strategy. One candidate hippocampus-independent strategy is the application of a conditional if-then rule to each of the six trial types, for example, “if left diagonal-right diagonal-blank go left (trial type 1 in dPAL; see Fig. 1a)” or “if right diagonal-vertical-blank go middle (trial type 3 in dPAL)” (conditional learning of this type in the touch screen is unaffected by fornix lesion-induced dysfunction of the hippocampus; Bussey et al. (2000)). For the mice using this strategy, the sPAL probe, in which e.g. left diagonal-right diagonal-blank is replaced by left diagonal-left diagonal-blank, should disrupt such a strategy. However, this challenge, while decreasing performance in both groups, had no differential effect on the performance of the dHp-lesioned mice.

A related idea is that in the absence of dHp, the task is still solved using an object-location strategy, but by involving other structures capable of mediating this type of learning. One candidate is the ventral hippocampus (vHp). Over extended training, vHp neurons can gradually discriminate spatial contexts in a context-guided object-association task very similar to cdPAL (Komorowski et al. 2013). The effects of vHp dysfunction on dPAL and cdPAL remain to be explored. Another possibility is that the effects of post-acquisition muscimol were due to state dependency of retrieval; i.e. muscimol infusions altered the animals’ internal context such that a mismatch between pre- and post-infusion context hampered context-assisted retrieval mechanisms. It is difficult to rule out such explanations in this type of experiment; however, in this case, given the magnitude of the effects of muscimol, it is difficult to imagine that they were entirely due to state dependency.

Another unanswered question is as follows: In cdPAL, why did adding contexts to discriminate A+ vs. B− from A− vs. B+ not render acquisition of the task more hippocampus dependent? Such context-dependent discriminations have been reported to be impaired by hippocampal dysfunction (Gaffan and Harrison 1989; Lee and Solivan 2008). Furthermore, neurons in the hippocampus discriminate objects in distinct contexts, in a task very similar to cdPAL (Komorowski et al. 2013). However, in the study of Komorowski, this was the vHp, and in the present study, the vHp was spared. Conceivably, it is the vHp, not the dHp, that mediates context-dependent learning (see Nadel et al. 2013; Strange et al. 2014 for reviews). Another possibility is that structures other than the hippocampus are required to discriminate very similar contexts like those used here. Indeed, hippocampal place cells are not good at discriminating chambers that are perceptually similar (Spiers et al. 2015). Entorhinal cortex is another possibility (Hafting et al. 2005). Alternatively, the contexts could be being discriminated by the mice in egocentric coordinates, e.g. left and right relative to the animal. However, it should be re-stated that it was only the acquisition of the task in the presence of the dHp lesion that was relatively spared; as discussed above, post-acquisition performance was strongly impaired by dHp inactivation, suggesting that acquisition of the task under normal circumstances does involve the dHp.

Finally, the present results may have theoretical implications. It is often assumed that discrimination learning that is learned incrementally over a number of trials—of which rodent PAL is an example—is performed using a habit system that is independent, or becomes independent, of the hippocampus (Zola and Squire 2000). The finding that hippocampus lesions only mildly impair acquisition of the task could be taken as evidence in favour of this idea. However, once the task was acquired with an intact hippocampus, hippocampal inactivation severely disrupted task performance, showing that although the task was learned incrementally over a number of trials, its performance still depended on the hippocampus. This adds to other evidence showing that just because a task is learned incrementally over a number of trials, it does not mean that its performance is independent of structures in the medial temporal lobe (Bussey et al. 2003).

## Conclusions

The present study has shown that (1) performance of dPAL in the mouse is highly sensitive to dHp dysfunction, as it is in the rat; (2) pre-acquisition lesions of dHp have little effect on acquisition, showing that in the absence of a dHp, other mechanisms or structures can compensate; and (3) the new context-dependent cdPAL task is also highly sensitive to dHp dysfunction, and like dPAL, cdPAL acquisition is only mildly affected by lesions of dHp. Other neural systems underlying the cdPAL task remain to be elucidated, but this task may provide a way to probe other systems such as the vHp. Finally, this study has used, for the first time, a number of behavioural challenges that can be added to the PAL tasks, including increasing the number of objects to be mapped on to the locations in dPAL or spatial contexts in cdPAL and degrading the visual objects with noise, which extend the utility of these tasks.
